# Altered Tim-1 and IL-10 Expression in Regulatory B Cell Subsets in Type 1 Diabetes

**DOI:** 10.3389/fimmu.2021.773896

**Published:** 2021-12-21

**Authors:** Yikai Liu, Zhiying Chen, Junlin Qiu, Hongzhi Chen, Zhiguang Zhou

**Affiliations:** National Clinical Research Center for Metabolic Diseases, Key Laboratory of Diabetes Immunology, Ministry of Education, and Department of Metabolism and Endocrinology, The Second Xiangya Hospital of Central South University, Changsha, China

**Keywords:** Tim-1, IL-10, T1D (type 1 diabetes), regulatory B (Breg) cells, autoantibody

## Abstract

**Background:**

Type 1 diabetes (T1D) is an autoimmune disease with a complex aetiology. B cells play an important role in the pathogenesis of T1D. Regulatory B cells (Bregs) are a subset of B cells that produce and secrete the inhibitory factor interleukin-10 (IL-10), thereby exerting an anti-inflammatory effect. It was recently discovered that T-cell immunoglobulin mucin domain 1 (Tim-1) is essential for maintaining Bregs function related to immune tolerance. However, the detailed understanding of Tim-1^+^ Bregs and IL-10^+^ Bregs in T1D patients is lacking. This study aimed to characterize the profile of B cell subsets in T1D patients compared with that in controls and determine whether Tim-1^+^ Bregs and IL-10^+^ Bregs play roles in T1D.

**Materials and Methods:**

A total of 47 patients with T1D, 30 patients with type 2 diabetes (T2D) and 24 healthy controls were recruited in this study. Flow cytometry was used to measure the levels of different B cell subsets (including B cells, plasmablasts, and Bregs) in the peripheral blood. Radiobinding assays were performed to detect the antibody titres of T1D patients. In addition, the correlations between different B cell subsets and patient parameters were investigated.

**Results:**

Compared with healthy controls, differences in frequency of Tim-1^+^ Bregs were significantly decreased in patients with T1D (36.53 ± 6.51 *vs*. 42.25 ± 6.83, *P*=0.02^*^), and frequency of IL-10^+^ Bregs were lower than healthy controls (17.64 ± 7.21*vs*. 24.52 ± 11.69, *P*=0.009^**^), the frequency of total Bregs in PBMC was also decreased in patients with T1D (1.42 ± 0.53*vs*. 1.99 ± 0.93, *P*=0.002.^**^). We analyzed whether these alterations in B cells subsets were associated with clinical features. The frequencies of Tim-1^+^ Bregs and IL-10^+^ Bregs were negatively related to fasting blood glucose (FBG) (*r*=-0.25 and -0.22; *P*=0.01^*^ and 0.03^*,^ respectively). The frequencies of Tim-1^+^ Bregs and IL-10^+^ Bregs are positively correlated with fast C-peptide (FCP) (*r*=0.23 and 0.37; *P*=0.02^*^ and 0.0001^***^, respectively). In addition, the frequency of IL-10^+^ Breg was also negatively related to glycosylated haemoglobin (HbA1c) (*r*=-0.20, *P*=0.04^*^). The frequencies of Tim-1^+^ Bregs, IL-10^+^ Bregs and Bregs in T2D patients were reduced, but no statistically significant difference was found between other groups. Interestingly, there was positive correlation between the frequencies of Tim-1^+^ Bregs and IL-10^+^ Bregs in T1D (*r*=0.37, *P*=0.01^*^). Of note, it is worth noting that our study did not observe any correlations between B cell subsets and autoantibody titres.

**Conclusions:**

Our study showed altered Tim-1 and IL-10 expression in regulatory B cell in T1D patients. Tim-1, as suggested by the present study, is associated with islet function and blood glucose levels. These findings indicate that Tim-1^+^ Bregs and IL-10^+^ Bregs were involved in the pathogenesis of T1D.

## Introduction

Type 1 diabetes (T1D) is an autoimmune disease mediated by T cells that selectively destroys insulin-producing β cells (1). It is generally believed that B cells, an important type of antigen-presenting cell that expresses costimulatory signalling molecules, participate in the activation and expansion of autoreactive CD4^+^ T cells and CD8^+^ cytotoxic T cells, thereby contributing to the occurrence of T1D (2).

In animal model experiments, B cells have been shown to infiltrate the islets of young nonobese diabetic (NOD) mice and mediate the autoimmune response that causes β cell destruction (3). In clinical trials, the use of rituximab (an anti-CD20 monoclonal antibody) was shown to effectively postpone the decline in pancreatic β cell function in patients with T1D (4). These results demonstrated that B lymphocytes play an indispensable and important role in the pathogenesis of T1D. This finding opened up a new way to explore T1D. Multiple subsets of B cells may be involved in immune homeostasis and prevent autoimmunity. Regulatory B cells (Bregs) are characterized by a cell-surface CD19^+^ CD24^hi^ CD38^hi^ phenotype (5). It has recently been established that Bregs have regulatory functions in infections, allergies, transplantation and autoimmune diseases (6). Bregs down-regulate the immune response by producing the inhibitory cytokine interleukin-10 (IL-10). Therefore, Bregs play important roles in immune tolerance. IL-10 is an anti-inflammatory cytokine mainly produced by Bregs. It regulates cell growth and differentiation and participates in inflammatory and immune responses. This cytokine is currently recognized as an immunosuppressive factor (7). T cell Ig and mucin domain (Tim-1), a transmembrane glycoprotein, has been identified as one of the three human Tim family members (Tim-1, Tim-3, and Tim-4) (8). Tim-1 was previously reported to be expressed and function in T cells; in addition to its role in T cells, Tim-1 signalling on B cells was recently shown to play roles in maintaining the stability of the immune system and inhibiting autoimmune diseases (9). Researchers have generated Tim-1 mutant mice [Tim-1 (Δmucin)], in which the mucin domain of Tim-1 is genetically deleted. The function of Bregs in these mutant mice to produce IL-10 is defective (10).

There is a growing list of autoimmune diseases that are associated with Breg dysregulation, including multiple sclerosis (MS), systemic lupus erythaematosus (SLE) and rheumatoid arthritis (RA) (11–13). In addition, many studies have demonstrated that there are negative correlations between the frequency of Bregs and the activity of some diseases. However, little is known about the frequency of Tim-1 or IL-10 in Bregs in patients with T1D. Therefore, the aim of this study was to compare the frequencies of Tim-1^+^ Bregs and IL-10^+^ Bregs in patients with T1D to those in healthy controls.

## Materials and Methods

### Patients and Controls

Forty-seven patients with T1D (28 males and 19 females; mean age=30.79 ± 9.05), 30 T2D (17 males and 13 females; mean age=46.63 ± 9.43) and 24 healthy controls (15 males and 9 females; mean age=33.33 ± 6.20) were enrolled in this study at the Second Xiangya Hospital. The diagnosis of T1D was made based on the World Health Organization (WHO) criteria (14). The diagnosis of T1D was based on acute onset ketosis or ketoacidosis, and all patients diagnosed with T1D required immediate insulin replacement therapy. At least one classic pancreatic islet autoantibody (anti-GAD antibodies [GADA], anti-zinc transporter 8 antibodies [ZnT8A] or anti-insulinoma-associated protein-2 antibodies [IA-2A]) was positive, or patients exhibited impaired C-peptide secretion. The diagnostic criteria for T2D is a typical history of hyperglycaemia according to WHO criteria, islet autoantibodies are negative and insulin therapy is not needed immediately. 24 healthy controls had no history of autoimmune diseases, pregnancy, or malignant diseases. All patients were confirmed to be negative for chronic infection disease, allergic or cancer and did not receive any immunosuppressive medication. Our study was approved by the ethics committee of Second Xiangya Hospital, Central South University. All subjects voluntarily joined this study and provided informed consent. The characteristics of all subjects are summarized in **Table 1**.

**Table 1:** General characteristics of study subjects.

	Type 1 diabetes	Type 2 diabetes	Healthy control
Sex(male/female)	28/19	17/13	15/9
Age (years)	30.79 ± 9.05^****^	46.63 ± 9.43^####^	33.33 ± 6.20
TG (mmol/L)	0.81(0.54-1.17)^****^	1.66(1.18-2.43)	1.12 (0.75-1.77)^Δ^
TC (mmol/L)	4.20 ± 0.98	4.72 ± 0.95	4.36 ± 0.80
LDL-C (mmol/L)	2.35 ± 0.71^**^	2.95 ± 0.83	2.56 ± 0.82
HDL-C (mmol/L)	1.56 ± 0.48^***^	1.17 ± 0.21^#^	1.45 ± 0.49
FBG (mmol/L)	7.06(5.43-10.37)	8.33(6.52-11.12)^####^	4.80(4.45-5.18)^ΔΔΔΔ^
HbA1c(%)	7.45 ± 1.45^*^	8.16 ± 1.74^####^	5.31 ± 0.34^ΔΔΔΔ^
FCP (pmol/L)	39.30 (16.5-154.1)^****^	436.9(252.4-567.0)	556.8(332.9-734.9)^ΔΔΔΔ^
Duration (months)	40.66 ± 24.69^*^	68.47 ± 50.46	NA
GADA	27/47 (57.4%)	NA	NA
IA-2A	11/47 (23.4%)	NA	NA
ZnT8A	11/47 (23.4%)	NA	NA

Data are expressed as (n) for qualitative data, the mean ± standard deviation for parametric data and the median (interquartile ranges) for nonparametric data. NA, not applicable.

^*^P < 0.05 compared to T2D, ^**^P < 0.01 compared to T2D, ^***^P < 0.001 compared to T2D, ^****^P < 0.0001 compared to T2D.

^#^P < 0.05 compared to healthy control, ^####^P < 0.0001 compared to healthy control.

^Δ^P < 0.05 compared to T1D, ^ΔΔΔΔ^P < 0.0001 compared to T1D.

### Peripheral Blood Mononuclear Cell (PBMC) Isolation

Venous blood samples (5 mL) were collected from each subject into heparinized sodium tubes, and PBMCs were isolated by Histopaque-1077 (Sigma-Aldrich, St. Louis, MO, USA) density gradient centrifugation. After centrifugation at 800 rcf for 23 min, PBMC was separated from the whole blood.

### Islet Autoantibody Assays

Radiobinding assays for GADA, ZnT8A and IA-2A were carried out as previously described (15, 16). Positive test results for GADA, IA-2A or ZNT8A were defined as 18.5 units/mL, 3.3 units/mL (WHO unit), and 0.011 (ZnT8A index), respectively.

### Glycosylated Haemoglobin (HbA1c) and β Cell Function

HbA1c levels were measured by liquid chromatography (Tosoh Corporation, Tokyo, Japan). Fasting C-protein (FCP) was measured by a chemiluminescence method (Siemens, Munich, Germany).

### Flow Cytometry

For intracellular staining of cytokines, PBMCs were stimulated with 1 μl/ml cell activation cocktail (BioLegend, San Diego, CA, USA) and 1 μl/ml monensin (BioLegend, San Diego, CA, USA) for 6 hours. PBMCs were harvested and stained with fixable viability stain (BD, Franklin Lakes, NJ, USA) and various monoclonal antibodies (anti-CD19-FITC, anti-CD24-PE, anti-CD38-APC, and anti-IL-10-PerCP-Cy5.5) (BD, Franklin Lakes, NJ, USA). Immunofluorescence staining for flow cytometric analysis was performed following the manufacturer’s instructions.

**Figure 1:**
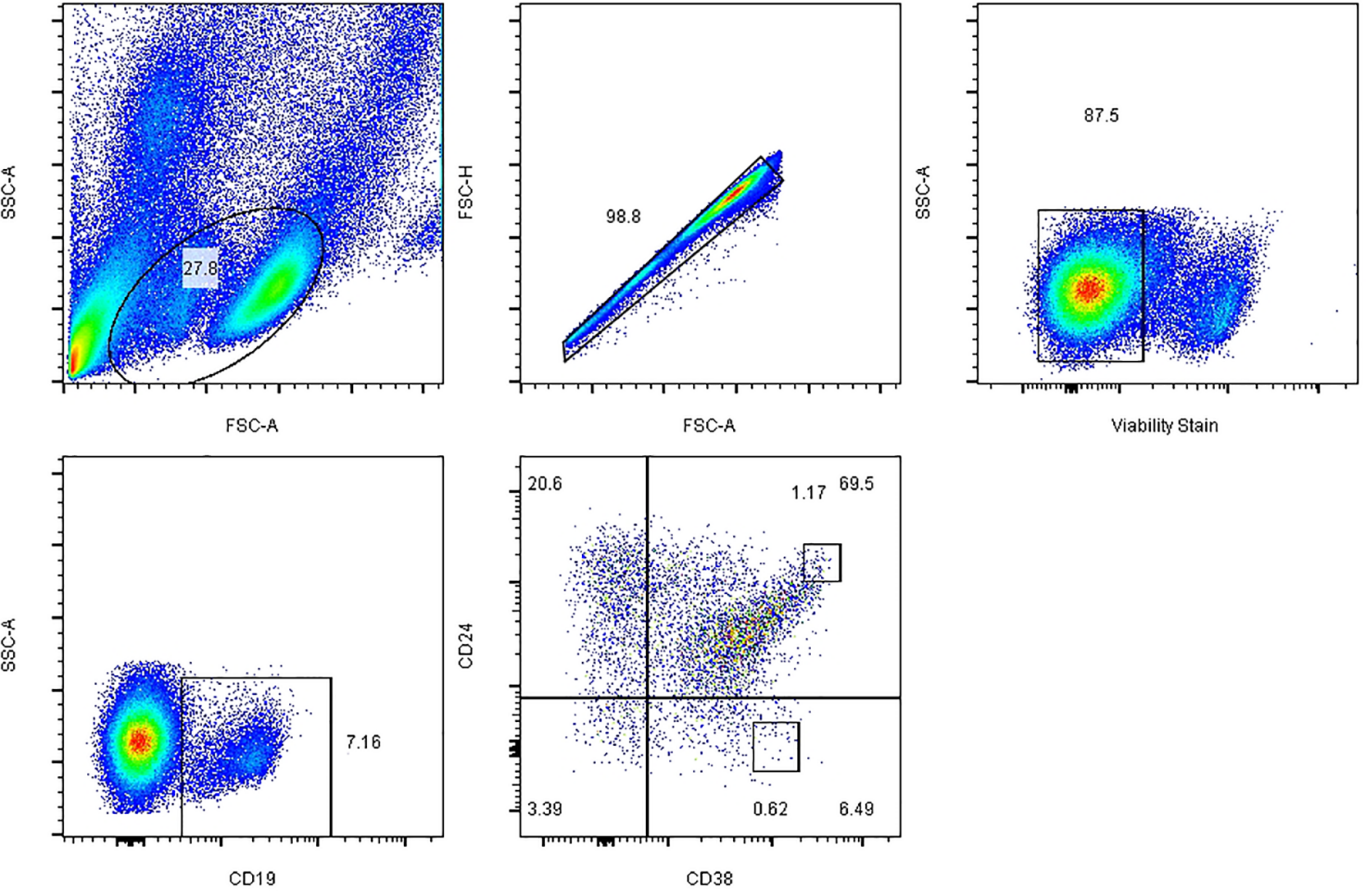
Representative plots and the gating strategy for fluorescence-activated cell sorting analysis of B cell subsets. Initial CD19^+^ B cells were sequentially gated on total lymphocytes, single cells, live cells and CD19^+^ B cells, and the expression of Breg and plasmablast markers on CD19^+^ cells was further analysed. FSC-A, forward scatter area; FSC-H, forward scatter height; SSC-A, side scatter area.

To determine Tim-1^+^ Breg frequencies, PBMCs were stained for CD19, CD24, CD38, and Tim-1 (anti-CD19- FITC, anti-CD24-FE, anti-CD38-APC, and anti-Tim-1-PerCP-Cy5.5) as surface markers. Data were collected on a Canto flow cytometer (BD, Franklin Lakes, NJ, USA) and analysed with FlowJo X software (Tree Star, Ashland, OR, USA). Dead cells were excluded from flow cytometric analysis on the basis of their forward- and side-light scatter properties and staining with Fixable Viability Stain 780 (BD, Franklin Lakes, NJ, USA). The gating strategies for all B cell subsets are described in **Figure 1**. PBMCs were isolated, and CD19^+^ B cell subsets were defined as follows: CD19^+^ (total B cells), CD19^+^ CD24^hi^ CD38^hi^ (Bregs), and CD19^+^ CD24^−^ CD38^hi^ (plasmablasts). The results are expressed as the percentages of Tim-1^+^ Bregs and IL-10^+^ Bregs (17).

### Statistical Analysis

All statistical analyses were performed with IBM SPSS version 24 (IBM Corporation, Chicago, IL, USA) and GraphPad Prism 8 (GraphPad Software, San Diego, CA, USA). In the correlation analysis, the frequency of various B cell subsets was logarithmically transformed, the clinical features conforming to the normal distribution were analyzed by Pearson correlation, and the non-parametric data was analyzed by Spearman. Data are presented as the mean ± standard deviation of the mean. Continuous variables were compared by one-way ANOVA. *P* < 0.05 was considered to indicate a significant difference.

## Results

### Demographic and Clinical Characteristics

One hundred and one participants were enrolled in our study, and **Table 1** summarizes the features of the subjects in each group. The T1D patients included 28 males and 19 females. The mean duration of T1D after diagnosis was 40.66 ± 24.69 months. T2D patients were older than T1D and healthy control groups, and the duration of diabetes was longer than T1D (68.47 ± 50.46 months). Compared with other groups, T2D have higher levels of triglycerides(TG), total cholesterol(TC), LDL cholesterol(LDL-C) and HDL cholesterol (HDL-C). The fasting blood glucose levels of the two groups of diabetic patients were similar, but the HbA1c of T2D was higher than that of T1D. Of note, the FCP of T1D was significantly lower than that of T2D and healthy controls. There was no statistical difference in gender or age among the three groups.

### Frequencies of B Cell Subsets Among T1D, T2D and Healthy Controls

**Figure 2:**
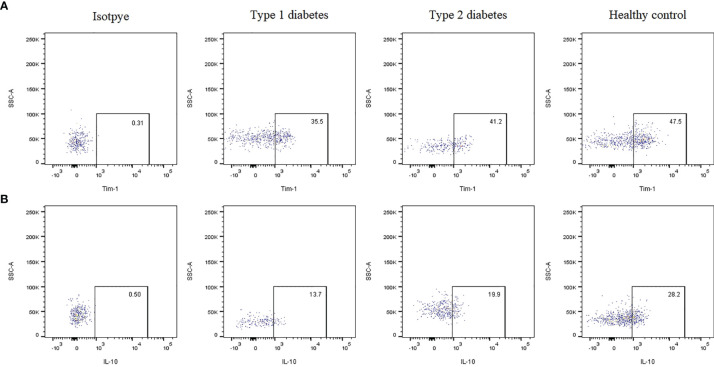
Representative dot plots showing the expression of Tim-1 or IL-10 by Bregs in T1D, T2D patients and healthy controls evaluated using flow cytometric analysis. **(A)** Frequency of Tim-1^+^ Bregs in various groups. **(B)** Frequency of IL-10^+^ Bregs in various groups.

The gating strategy for B cell subset quantification was based on CD19, CD24 and CD38 frequency, as shown in **Figure 1**. Flow cytometric analysis indicated that Tim-1^+^ Bregs were significantly decreased in T1D patients (36.53 ± 6.51 *vs*. 42.25 ± 6.83, *P*=0.02^*^) (**Figure 2A**). In contrast, the frequency of IL-10^+^ Bregs was lower in T1D patients than that of healthy controls (17.64 ± 7.21 *vs*. 24.52 ± 11.69, *P*=0.009^**^) (**Figure 2B**). The frequency of Tim-1^+^ Bregs and IL-10^+^ Bregs in T2D patients was amid that of T1D and healthy controls (38.11 ± 11.30, 20.67 ± 9.19, respectively). But there was no statistical difference between T2D and other two groups(*P*>0.05 for both).

**Figure 3:**
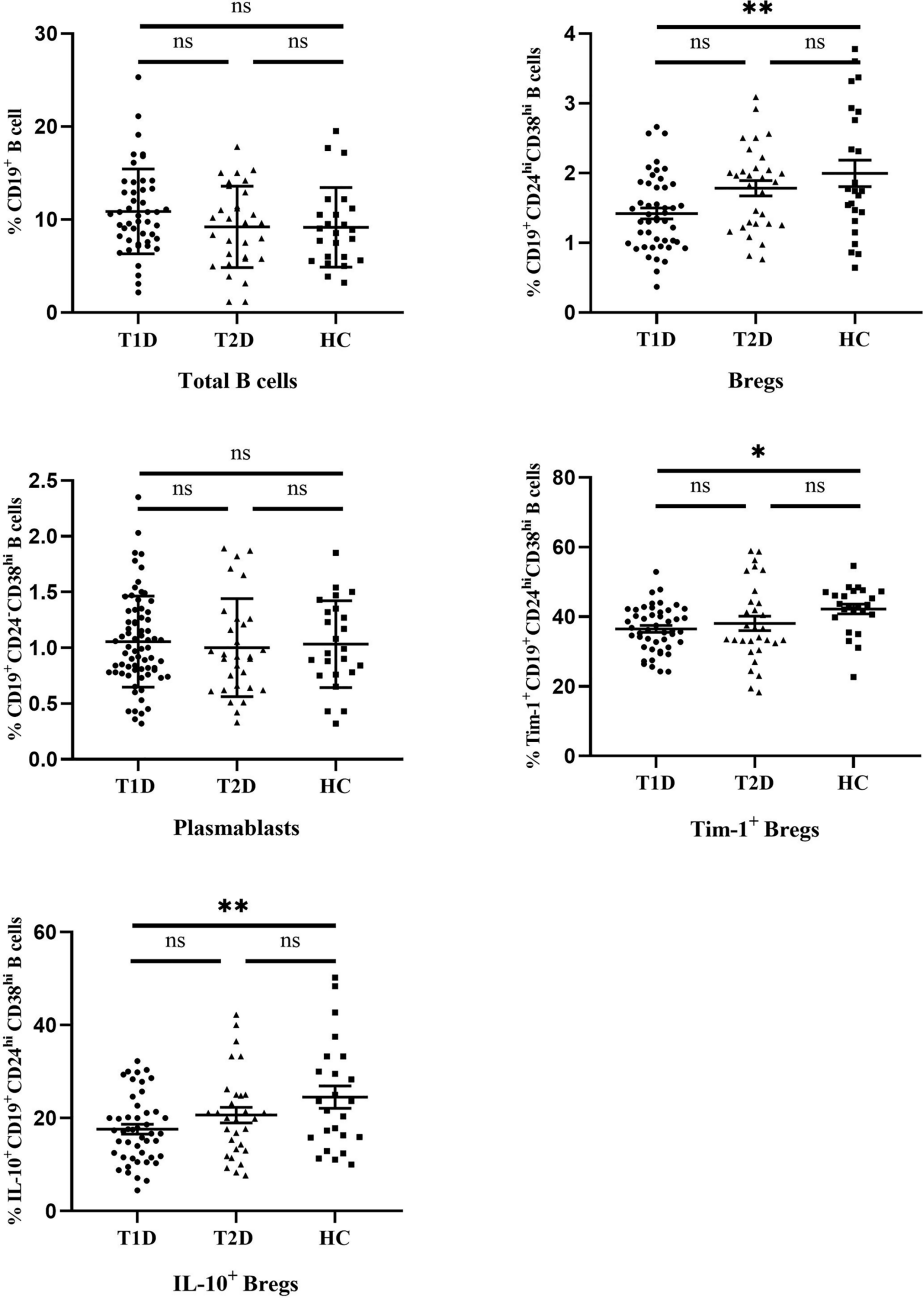
The frequencies of different B cell subsets in T1D, T2D patients and healthy controls. Horizontal lines show the medians. T1D, type 1 diabetes; T2D, type 2 diabetes; HC, healthy control. ns, no significant; ^*^ *P* < 0.05; ^**^ *P* < 0.01.

The differences in the frequencies of different B cell subsets are summarized in **Figure 3**. There were no significant differences in the frequencies of total B cells (CD19^+^) or plasmablasts (CD19^+^ CD24^-^ CD38^hi^) between T1D, T2D and controls (10.87 ± 4.56 and 1.07 ± 0.42 in T1D patients, 9.17 ± 4.3 and 1.00 ± 0.44 in T2D patents, 9.20 ± 4.4 and 1.03 ± 0.39 in healthy controls for total B cells and plasmablasts, respectively). Interestingly, Bregs (CD19^+^ CD24^hi^ CD38^hi^) were significantly lower in T1D patients than that of healthy controls (*P*=0.002^**^). The mean frequency of Bregs in the T1D patients was 1.42 ± 0.53, while the value in the controls was 1.99 ± 0.93. Compared with healthy controls, the frequency of Bregs in T2D patients showed a decreasing trend (1.78 ± 0.61), but no significant difference was found between the T2D and other two groups.(*P*=0.06 compared to T1D, *P* =0.48 compared to controls, respectively).

### Correlations Between the Frequencies of B Cell Subsets and Clinical Features of All the Study Subjects

**Figure 4:**
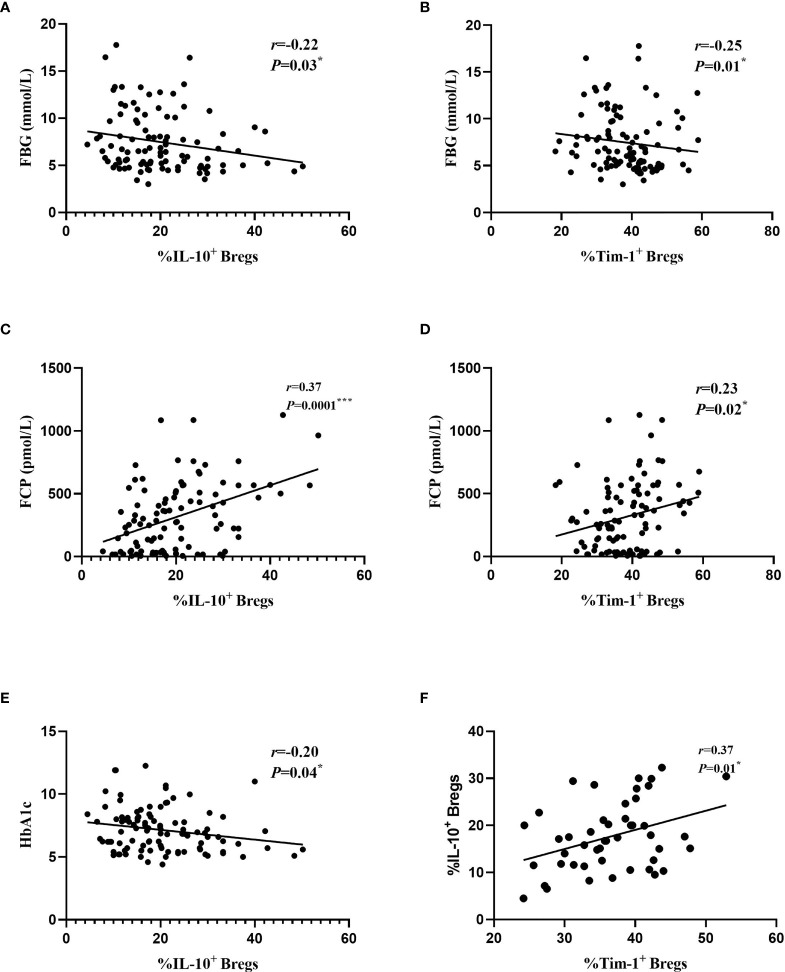
Relationships between different B cell subsets and clinical data from all the study subjects. **(A)** Correlations between IL-10^+^ Bregs and FBG. **(B)** Correlations between Tim-1^+^ Bregs and FBG. **(C)** Correlations between IL-10^+^ Bregs and FCP. **(D)** Correlations between Tim-1^+^ Bregs and FCP. **(E)** Correlations between IL-10^+^ Bregs and HbA1c. **(F)** Correlations between IL-10^+^ Bregs and Tim-1^+^ Bregs in T1D. Each point represents an individual patient with T1D, T2D and healthy controls. ^*^ *P* < 0.05. ^***^ *P* < 0.001.

Given that B cells play an important role in the development of diabetes, we next determined whether the changes in various B cell subsets in all participants had any correlation with clinical features (**Table 2**). The frequencies of Tim-1^+^ Bregs and IL-10^+^ Bregs were negatively correlated to fasting blood glucose (FBG) (*r*=-0.25 and -0.22; *P*=0.01^*^ and 0.03^*,^ respectively). and positively correlated with FCP (*r*=0.23 and 0.37; *P*=0.02^*^ and 0.0001^***^, respectively). Moreover, the frequency of IL-10^+^ Bregs was also negatively related to glycosylated haemoglobin (HbA1c) (*r*=-0.20, *P*=0.04^*^). In addition, we also found a positive correlation between Tim-1^+^ Bregs and IL-10^+^ Bregs in T1D patients (*r*=0.37, *P*=0.01^*^) (**Figure 4**)

**Table 2:** Correlations between the frequencies of B cell subsets and clinical features of all the study subjects.

	CD19^+^ B cells		Bregs		Plasmablasts		Tim-1^+^ Bregs		IL-10^+^ Bregs	
	*r*	*P* value	*r*	*P* value	*r*	*P* value	*r*	*P* value	*r*	*P* value
Male^a^	0.04	0.68	0.02	0.81	-0.06	0.56	-0.13	0.19	-0.01	0.89
Age	-0.18	0.07	0.15	0.11	-0.17	0.08	0.10	0.33	0.76	0.45
TG^a^	-0.14	0.16	0.08	0.43	-0.10	0.34	0.11	0.29	0.15	0.13
TC	-0.07	0.49	0.11	0.28	-0.15	0.14	0.05	0.63	0.01	0.95
LDL-C	-0.10	0.35	0.18	0.07	-0.07	0.50	0.02	0.86	-0.02	0.83
HDL-C	0.14	0.15	-0.12	0.25	-0.11	0.28	0.01	0.91	-0.00	0.99
FBG^a^	-0.08	0.44	-0.19	0.06	-0.12	0.23	-0.25	0.01^*^	-0.22	0.03^*^
HbA1c	-0.01	0.93	-0.14	0.16	-0.08	0.44	-0.15	0.12	-0.20	0.04^*^
FCP^a^	-0.18	0.07	0.17	0.08	-0.02	0.86	0.23	0.02^*^	0.37	0.00^***^
GADA^a^	-0.09	0.55	0.19	0.20	0.26	0.07	-0.01	0.92	-0.10	0.48
IA-2A^a^	-0.01	0.93	-0.07	0.62	0.25	0.09	0.14	0.34	0.10	0.48
ZnT8A^a^	0.19	0.20	-0.07	0.63	0.02	0.91	-0.05	0.73	-0.07	0.64

Correlation analyses between various B cell subset frequencies (after log transformation) and clinical features were performed by Pearson test.

a compared by spearman correlations. ^*^ indicates significance (P < 0.05), ^***^ indicates significance (P < 0.001).

### Correlation Between Tim-1^+^ or IL-10^+^ Bregs With Autoantibodies in T1D Patients

**Figure 5:**
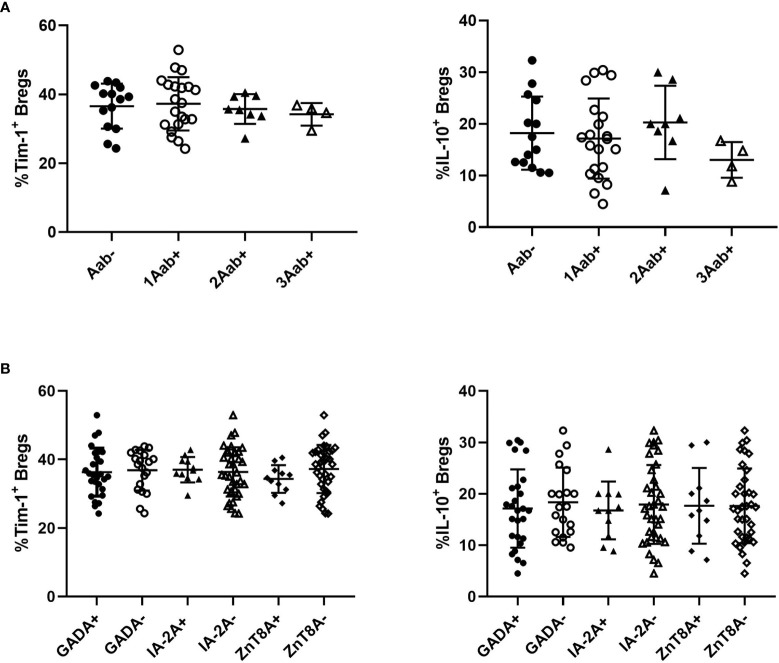
Relationships between Tim-1 or IL-10 expressed by Bregs and autoantibodies in T1D patients. **(A)** Correlations between the number of positive antibodies and the frequency of Tim-1^+^ or IL-10^+^ Bregs. **(B)** Correlations between the types of positive antibodies and the frequency of Tim-1^+^ or IL-10^+^ Bregs.

We then examined the relationships between Tim-1^+^ Bregs or IL-10^+^ Bregs and the studied autoantibodies. There were no significant differences in the frequencies of Tim-1^+^ Bregs or IL-10^+^ Bregs among T1D patients who were positive for one, two, or three of the antibodies (**Figure 5A**). Additionally, no difference in Tim-1^+^ Bregs or IL-10^+^ Bregs was observed in patients regardless of autoantibody testing results(**Figure 5B**).

## Discussion

In the present study, we investigated the frequencies of various B cell subsets in patients with T1D. Our study showed that the frequency of Bregs, Tim-1^+^ Bregs and IL-10^+^ Bregs in diabetic patients showed a declining trend, which was statistically significant in the T1D group. FBG and HbA1c respectively reflect the short- and long-term blood glucose level of diabetic patients, although the T2D had higher FBG and HbA1c levels compared with the T1D patients, the frequencies of Bregs, Tim-1^+^ Bregs and IL-10^+^ Bregs in the T2D appears not to be lower than that in the T1D. This discrepancy may be due to heterogeneous pathogenesis of the two diabetes groups. T1D is a chronic autoimmune disease that is mainly caused by the destruction of pancreatic islet β cells mediated by T lymphocyte. Impaired pancreatic islet β cells exhibits a decreased FCP levels (18). B cells are important antibody-producing cells, antigen-presenting cells, and expressing costimulatory signals involved in the activation of CD4^+^ and CD8^+^ T cells. The dysfunction of B cells has an important impact on the pathogenesis of type 1 diabetes. However, metabolic dysfunction and chronic inflammation are the main clinical symptoms in T2D patients (19). TG, TC, HDL-C, and LDL-C are known for common metabolic indicator (20). We did not identify any correlation between Breg subsets and these indices in all subjects. This result ties well with previously published research (21). All these results indicate that changes in B cell subsets may contribute to immune disorders. In addition, correlations between the frequencies of B cell subsets and clinical features were confirmed in all subjects. The frequencies of Tim-1^+^ and IL-10^+^ Bregs were negatively correlated with FBG and positively correlated with the FCP levels. The frequency of IL-10^+^ Bregs was also negatively correlated with HbA1c levels, However, neither Tim-1^+^ Bregs nor IL-10^+^ Bregs were related to the number and types of islet-related autoantibodies.

The Tim gene family contains three Tim proteins (Tim-1, Tim-3, and Tim-4). Tim-1 is a glycoprotein involved in the immune response. Tim-3 expression has been detected on innate and adaptive immune cells. Tim-4 is a natural ligand of Tim-1. Emerging evidence suggests that the binding of Tim-1 to Tim-4 is involved in T cell proliferation (22). However, whether Tim-3 or Tim-4 is expressed in Breg cells is not yet clear (23). Early studies found that Tim-1 is expressed in T cells participates in the activation of T cells and regulates the immune response of T helper cells (8). Recent studies revealed that Tim-1 also expressed in other immune cells, such as mast cells (24), Tregs and B cells (25, 26). Interestingly, Tim-1 signal transduction is necessary for the maintenance and induction of IL-10 in Bregs (9). Tim-1-mutant B cells exhibit a reduced apoptotic cells binding capacity and incapable to produce IL-10 effectively (25). Aravena et al. evaluated the frequency of Tim-1 in different B cell subpopulations. Their study presented that human transitional B cells, as a phenotype associated with Bregs, have the highest content of Tim-1^+^ cells, and most Tim-1^+^ Bregs also express IL-10 (27). It is worth noting that in their study, the frequency of Tim-1^+^ Bregs in systemic sclerosis patients was lower than that in healthy people. At the same time, the competence of the Tim-1^+^ Bregs in patients was compromised. All these results indicate that Tim-1 is essential for regulating immune tolerance in Bregs.

As a major immunomodulatory cytokine, IL-10 affects many cells of the immune system (28). It has a powerful anti-inflammatory function, mainly targeting antigen-presenting cells (APCs) such as monocytes and macrophages. It also inhibits the release of pro-inflammatory cytokines. IL-10 can reduce the expression of major histocompatibility antigen II (MHC II) on the surface of monocytes. Thus it reduces antigen presentation by these cells, downregulates the activity of T lymphocytes, and inhibits the activation, migration and adhesion of inflammatory cells. At the same time, IL-10 can also inhibit the synthesis and release of inflammatory factors in Th cells (29). Moreover, IL-10 can directly act on T cells to inhibit their proliferation and cytokine production. Thereby reducing inflammation. Meanwhile, IL-10 plays an important role in promoting the activation and proliferation of human B cells (7). The immunomodulatory properties of Bregs depend on the production of IL-10. The importance of IL-10 has been confirmed both *in vivo* and *in vitro*. However, due to the low frequency of Bregs and the lack of specific transcription markers, research on how Bregs are induced is still limited.

Deng et al. illustrated that changes in the phenotypes of B cell subsets are related to the onset of autoimmune diabetes. The frequencies of B cell subsets that produce IL-10 (defined as B10) in T1D patients were found to be reduced, and the frequency of B10 cells was positively correlated with FCP and negatively correlated with HbA1c (21). Wang et al. showed that the frequencies of CD24^hi^ CD38^hi^ B cells in T1D patients were reduced. In addition, the frequency of CD24^hi^ CD38^hi^ B cells was negatively correlated with HbA1c (30) which was similar to the current study. Our data showed that compared with healthy controls, T1D patients exhibit decreased levels of Tim-1^+^ Bregs, IL-10^+^ Bregs and total Bregs. Moreover, the frequency of IL-10^+^ Bregs was positively correlated with Tim-1^+^ Bregs in T1D patients.

Diabetes-related autoantibodies can be used to assess the progression of T1D (31), and the titres of autoantibodies also seem to affect the complications of T1D (32, 33). High-titre antibodies are more likely to be accompanied by other autoimmune diseases or autoimmune-related antibody positivity. Islet autoantibodies (including GADA, IA2A and ZnT8A) can predict the risk of T1D and improve the sensitivity of diagnosing T1D patients (34, 35). Nevertheless, we did not find a correlation between B cell subsets and islet-related autoantibody in the current study. Considering that there are differences in measured GADA titres among different laboratories, it is difficult to set a threshold for distinguishing high and low levels. Furthermore, GADA titres may also be related to the age, disease course, and medication level of the patient.

Overall, our work suggests that the frequencies of Bregs, Tim-1^+^ Bregs and IL-10^+^ Bregs in T1D patients are reduced, and the latter two were correlated with islet function and blood glucose levels. The current data indicates that alterations in B cell subsets are related to the pathogenesis of autoimmune diabetes. Our results highlight the potential of selective B cell-targeted therapy aimed at specifically eliminating pathogenic B cells but promoting Bregs in T1D patients, which is a method being reconsidered. Our work has certain limitations. First, it is a small sample size study. Attention may need be paid when extending the conclusion to large population. Second, patients in this study possess relatively long courses of disease. In view of the low islet-related antibody positivity rate, it is very likely that autoantibodies turned negative in some patients prior to this study, which may affect the correlation study of Tim-1 and autoantibodies. Third, our study was cross-sectional. We did not track our patients longitudinally, nor did we perform a cohort study. Long-term and large-scale experiments, such as experiments on B cell subset functional levels and metabolic pathway level changes, need to be performed in the future.

## Data Availability Statement

The original contributions presented in the study are included in the article/supplementary material. Further inquiries can be directed to the corresponding authors.

## Ethics Statement

The studies involving human participants were reviewed and approved by the ethics committee of Second Xiangya Hospital. The patients/participants provided their written informed consent to participate in this study.

## Author Contributions

YL searched the references, wrote the first draft of the paper, and revised the text. ZC and JQ critically revised the text and provided substantial scientific contributions. ZZ and HC proposed the project and revised the manuscript. All authors contributed to the article and approved the submitted version.

## Funding

This study was supported by the National Natural Science Foundation of China (grant numbers 81820108007 and 81970746) and the Natural Science Foundation of China (grant number 82000748).

## Conflict of Interest

The authors declare that the research was conducted in the absence of any commercial or financial relationships that could be construed as a potential conflict of interest.

## Publisher’s Note

All claims expressed in this article are solely those of the authors and do not necessarily represent those of their affiliated organizations, or those of the publisher, the editors and the reviewers. Any product that may be evaluated in this article, or claim that may be made by its manufacturer, is not guaranteed or endorsed by the publisher.
